# Conductive Metal–Organic Frameworks with Extra Metallic Sites as an Efficient Electrocatalyst for the Hydrogen Evolution Reaction

**DOI:** 10.1002/advs.202000012

**Published:** 2020-03-16

**Authors:** Hao Huang, Yue Zhao, Yimin Bai, Fumin Li, Ying Zhang, Yu Chen

**Affiliations:** ^1^ Key Laboratory of Applied Surface and Colloid Chemistry Ministry of Education School of Chemistry and Chemical Engineering Shaanxi Normal University Xi'an 710062 P. R. China; ^2^ Key Laboratory of Macromolecular Science of Shaanxi Province Shaanxi Key Laboratory for Advanced Energy Devices School of Materials Science and Engineering Shaanxi Normal University Xi'an 710062 P. R. China

**Keywords:** bimetallic sites, conjugated ligands, electrocatalysis, hydrogen evolution reaction, metal–organic frameworks

## Abstract

The 2D conductive metal–organic frameworks (MOFs) are expected to be an ideal electrocatalyst due to their high utilization of metal atoms. Exploring a new conjugated ligand with extra active metallic center can further boost the structural advantages of conductive MOFs. In this work, hexaiminohexaazatrinaphthalene (HAHATN) is employed as a conjugated ligand to construct bimetallic sited conductive MOFs (M2_3_(M1_3_∙HAHATN)_2_) with an extra M–N_2_ moiety. Density functional theory (DFT) calculations demonstrate that the 2D conjugated framework renders M2_3_(M1_3_∙HAHATN)_2_ a high electric conductivity with narrow bandgap (0.19 eV) for electron transfer and a favorable in‐plane porous structure (2.7 nm) for mass transfer. Moreover, the metal atom at the extra M–N_2_ moiety has a higher unsaturation degree than that at M–N_4_ linkage, resulting in a stronger ability to donate electrons for enhancing electroactivity. These characteristics endow the new conductive MOFs with an enhanced electroactivity for hydrogen evolution reaction (HER) electrocatalysis. Among the series of M2_3_(M1_3_∙HAHATN)_2_ MOF, Ni_3_(Ni_3_∙HAHATN)_2_ nanosheets with the optimal structure exhibit a small overpotential of 115 mV at 10 mA cm^−2^, low Tafel slope of (45.6 mV dec^−1^), and promising electrocatalytic stability for HER. This work provides an effective strategy for designing conductive MOFs with a favorable structure for electrocatalysis.

## Graphical abstract

Hexaiminohexaazatrinaphthalene (HAHATN) is employed as a conjugated ligand to introduce an extra M–N_2_ moiety for constructing M2_3_(M1_3_∙HAHATN)_2_ conductive metal–organic frameworks (MOFs) (M1 = Ni or Co or Cu; M2 = Ni or Cu). The extra metal atom in the M1–N_2_ moiety exhibits a higher unsaturated degree than that in traditional M2–N_4_ linkage, which remarkably enhances the electroactivity of conductive MOFs.

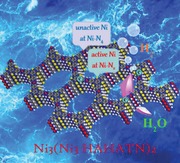

## 1 Introduction

Water electrolysis is a pivotal strategy to generate clean hydrogen, a promising alternative to fossil fuels.^[^ ^1^ ^]^ To date, the efficient electrocatalysts for hydrogen evolution reaction (HER) are mainly inorganic nanostructures.^[^ ^2^ ^]^ The active sites of these nanostructures for HER only exist on their surface and/or edge.^[^ ^3^ ^]^ The major unexposed metal atoms in the bulk phase are inert for HER, which strictly limits the metal atom utilization.^[^ ^4^, ^5^, ^6^ ^]^

Due to the exposed metal site, large surface area and structural controllability,^[^ ^7^, ^8^ ^]^ metal–organic frameworks (MOFs) are considered as preferred heterogeneous catalysts for various catalytic reactions.^[^ ^9^, ^10^, ^11^ ^]^ Particularly, the conductive subgroup of MOFs possesses a high ability of electron transfer beyond traditional MOFs. Consequently, the application of MOFs in electrocatalysis eventually becomes a reality.^[^ ^12^, ^13^ ^]^ Conductive MOFs consist of transition metal atoms and conjugated organic ligands by M–N_4_ linkages, which provide a fully π‐conjugated structure with exceptional electrical conductivity.^[^ ^14^ ^]^ Up to now, the conductive MOFs have been employed as efficient electrocatalysts for HER, oxygen evolution reaction, and oxygen reduction reaction.^[^ ^15^, ^16^, ^17^ ^]^ Although these conductive MOFs seem to possess the potential application for electrocatalysis due to the extraordinary structure, their electroactivity remains inferior to the practical application. The theoretical and experimental investigations have demonstrated that the metal atoms in the M–N_4_ linkages still remain the original oxidation state during the electrocatalytic process, which hardly shows effective activity for electrocatalysis.^[^ ^18^ ^]^ At present, hexaiminotriphenylene (HITP, Scheme S1, Supporting Information) and/or its analogs are usually employed as the conjugated organic ligands for conductive MOFs,^[^ ^19^, ^20^, ^21^, ^22^ ^]^ which barely provide other coordinated sites to be high active metal center for electrocatalysis. Thus, designing new conjugated organic ligand with extra coordinated sites to incorporate effective active centers should be the primary priority for improving electrocatalytic performance of conductive MOFs.

Hexaazatriphenylene (HATN, Scheme S2, Supporting Information) is a N‐containing tris(bidentate) polyheterocyclic ligand with electron‐deficient conjugated planar structure. The bidentate tertamine of HATN can coordinate various metal ions to form M_3_∙HATN with a two‐coordinated (M–N_2_) moiety.^[^ ^23^ ^]^ This structure can expose unoccupied positions of metal atom in a high content (≈14 at%), which are regarded as an efficient single‐atom center for catalysis.^[^ ^24^, ^25^ ^]^ HATN structure is also propitious to stabilize metallic ion with valence variation, which can serve as a key active intermediate for the electrocatalytic reaction.^[^ ^26^ ^]^ Since the structure of HATN are extreme controllable, various analogs have been developed as fundamental moieties to fabricate supramolecular systems, especially metallic‐supramolecular frameworks.^[^ ^27^, ^28^, ^29^ ^]^ As a result, HATN may be the optimal structure as a conjugated ligand to fabricate conductive MOFs. In this novel framework, the extra metal atoms, coordinated by bidentate tertamine, can serve as efficient active centers for electrocatalysis, similar to those of single‐atom catalysts.^[^ ^30^ ^]^ The fully conjugated structure and M–N_4_ linkages of conductive MOFs can offer strong guarantees for electron transfer, further improving electroactivity.^[^ ^31^, ^32^ ^]^ Additionally, the conductive MOFs, consisting of HATN‐based six‐member ring, have a larger structure with an expanded in‐plane pore (>2 nm), which can enhance the mass diffusion of small molecules in the framework.^[^ ^33^ ^]^ Thus, HATN‐based conductive MOFs may be ideal electrocatalysts with high activity. Unfortunately, HATN‐based conductive MOFs and their applications are rarely investigated so far.

In this work, hexaiminohexaazatrinaphthalene (HAHATN, Scheme S3, Supporting Information), an analog of HATN, is designed as the organic ligand to fabricate various bimetallic sited conductive MOFs with in‐plane mesoporous structures (2.7 nm). We present computational and experimental evidences for investigating the active sites in these conductive MOFs. Both theoretical and experimental results indicate the bidentate‐based metal site (M–N_2_) possesses higher activity than that of M–N_4_ linkage for HER. Ni_3_(Ni_3_∙HAHAT)_2_ nanosheets, the bimetallic sited conductive MOFs with optimal chemical compositions in this work, exhibit outstanding HER performances in alkaline solution, such as low overpotential of 115 mV at 10 mA cm^−2^, small Tafel plot of 45.6 mV dec^−1^, and excellent electrocatalytic stability. Therefore, employing HATN analog as conjugated organic ligand is an effective strategy to resolve the deficiency of traditional conductive MOFs in the field of electrocatalysis, which has extraordinary significance for designing and synthesizing of next‐generation conductive MOF‐based electrocatalysts.

## 2 Results and Discussion

**Scheme 1:**
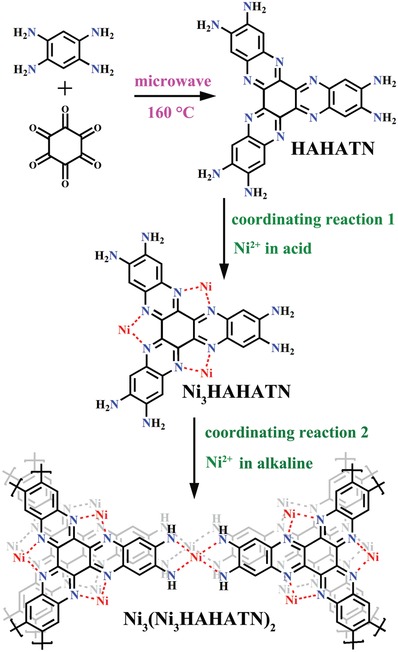
Synthetic diagram of conductive Ni_3_(Ni_3_∙HAHATN)_2_ MOFs.

Various nickel precursors including Ni‐based MOFs are usually used to construct efficient HER electrocatalysts due to their low cost and high activity, including Ni‐based MOFs.^[^ ^34^, ^35^ ^]^ In this work, we employ Ni_3_∙HAHATN as a conjugated ligand to construct conductive Ni_3_(Ni_3_∙HAHATN)_2_ MOFs via a synthetic step and two consecutive coordination reactions (**Scheme** **1**). The products in each synthetic process were preliminarily confirmed through nuclear magnetic resonance (NMR) and Fourier transform infrared (FTIR) technology (Figures S1–S5, Supporting Information). Similar to the traditional Ni_3_(HITP)_2_, Ni–N_4_ structure in Ni_3_(Ni_3_∙HAHATN)_2_ is used as linkage to construct the conjugated framework. Unfortunately, Ni atom at Ni–N_4_ linkage is low‐active for the electrocatalytic reactions due to its invariant oxidation state during electrochemical process.^[^ ^18^ ^]^ Beyond the Ni–N_4_ linkage, Ni_3_(Ni_3_∙HAHATN)_2_ also possesses extra Ni–N_2_ moieties with higher unsaturation degree, which can exhibit better electroactivity for the electrocatalytic reactions.

**Figure 1:**
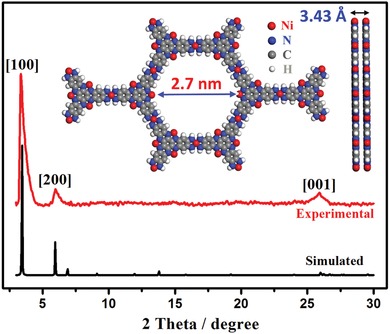
Experimental and simulated PXRD patterns of Ni_3_(Ni_3_∙HAHATN)_2_. The inset shows the optimization structure of Ni_3_(Ni_3_∙HAHATN)_2_ slab.

Powder X‐ray diffraction (PXRD) measurement reveals Ni_3_(Ni_3_∙HAHATN)_2_ has high crystallinity (**Figure** **1**). For clear clarification, we simulate the structure of Ni_3_(Ni_3_∙HAHATN)_2_. After geometry optimization, Ni_3_(Ni_3_∙HAHATN)_2_ shows a 2D metal–organic coordinated framework, constituted by a periodic hexagonal ring of six Ni_3_∙HAHATN moieties connected through Ni–N_4_ linkages (inset in Figure 1). An expanded mesoporous structure (2.7 nm) uniformly exists in the plane of Ni_3_(Ni_3_∙HAHATN)_2_ due to the larger ligand of Ni_3_∙HAHATN. As with P6/mmm symmetry, this type of MOFs has equivalent in‐plane lattice lengths approaching to 29.5 Å (*a* = *b*; α: 90°, β: 90°, γ  : 120°) with interlayer separation of ≈3.43 Å. The simulated PXRD result matches well with the experimental pattern, confirming the successful synthesis of Ni_3_(Ni_3_∙HAHATN)_2_. Consequently, the prominent peaks at 3.34° and 5.98° in PXRD can be separately assigned to (100) and (200) plane, respectively. A weak diffraction peak at 25.94° is corresponding to the (001) plane (3.43 Å), and inferring Ni_3_(Ni_3_∙HAHATN)_2_ can exist as few‐layered structure.

**Figure 2:**
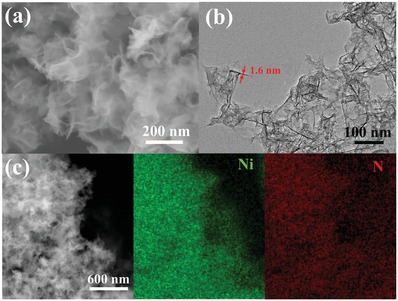
a) SEM, b) TEM and c) EDX Mapping images of Ni_3_(Ni_3_∙HAHATN)_2_ nanosheets.

Scanning electron microscopy (SEM) and transmission electron microscopy (TEM) were carried out to analyze the morphology and structure of Ni_3_(Ni_3_∙HAHATN)_2_. SEM images show Ni_3_(Ni_3_∙HAHATN)_2_ exhibits an obviously petaloid morphology in large‐scale, consisting of continuous thin‐layered nanosheets with abundant wrinkles (**Figure** **2**a). The interaction of these nanosheets brings an extensively hierarchical porous structure, and the size of pores is in range of dozens of nanometers. The anomalous porous morphology can efficiently increase electroactivity due to exposure of active centers and rapid mass diffusion for Ni_3_(Ni_3_∙HAHATN)_2_ nanosheets. N_2_ adsorption/desorption isotherm was carried out to investigate the porous feature of Ni_3_(Ni_3_∙HAHATN)_2_ nanosheets (Figure S6, Supporting Information). Pore size distribution curve shows the existence of two type mesoporous structures, and the wide peak at 46.3 nm is originated from hierarchical porous structure of the interlayer interaction. An obvious sharp peak at ≈2.6 nm is also observed, which is attributed to the in‐plane pores in keeping with the crystal structure of geometric optimization. Moreover, in this framework structure, the unsaturated nickel atom in Ni–N_2_ moiety is suspended in the in‐plane mesoporous structure, which is benefit to capture the proton. Similar to the SEM analysis, TEM image illustrates the Ni_3_(Ni_3_∙HAHATN)_2_ is made up of smooth silk‐like layered structures (Figure 2b). Generally, black‐line region in high‐resolution transmission electron microscopy (HRTEM) image is originated from the perpendicular nanosheets to support substrate, which can be used to estimate the thickness of nanosheets (Figure S7, Supporting Information).^[^ ^36^ ^]^ The width of the black‐line regions in this work is less than 1.6 nm, corresponding to the thickness of Ni_3_(Ni_3_∙HAHATN)_2_ nanosheets. Additionally, the analysis of atomic force microscopy (AFM) indicates that the thickness of Ni_3_(Ni_3_∙HAHATN)_2_ nanosheets is 1.6 nm (Figure S8, Supporting Information), in consistent with TEM characterization.

**Figure 3:**
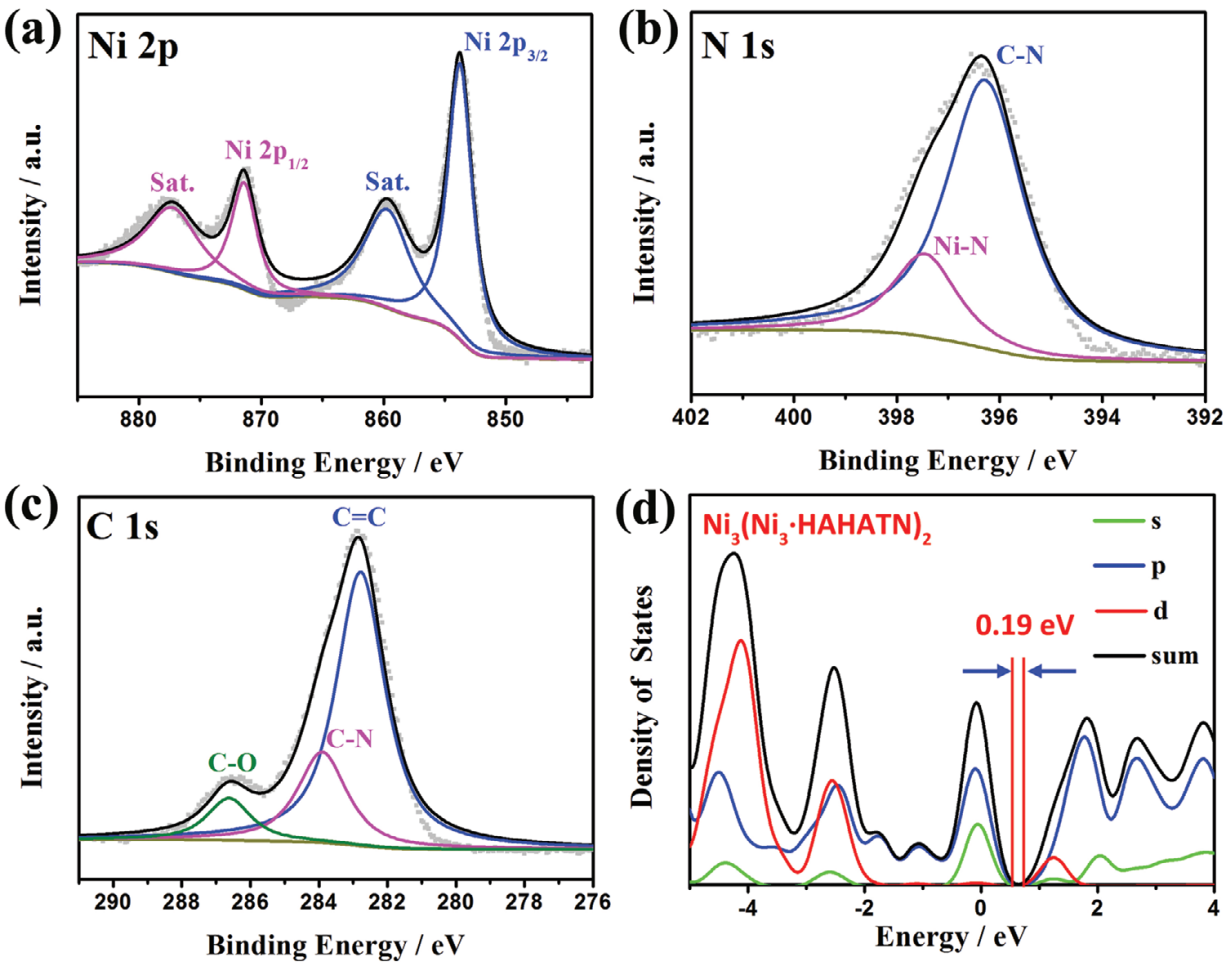
High‐resolution XPS spectra of a) Ni 2p, b) N 1s, and c) C 1s of Ni_3_(Ni_3_∙HAHATN)_2_. d) Calculated partial density of state (PDOS) of mono‐layered Ni_3_(Ni_3_∙HAHATN)_2_ slab.

The element composition and surface chemical state of Ni_3_(Ni_3_∙HAHATN)_2_ nanosheets were identified by energy dispersive spectroscopy (EDS) elemental mapping and X‐ray photoelectron spectroscopy (XPS) technologies. Mapping analysis reveals the uniform distribution of nickel and nitrogen element on Ni_3_(Ni_3_∙HAHATN)_2_ nanosheets (Figure 2c). XPS spectrum also proves the Ni_3_(Ni_3_∙HAHATN)_2_ nanosheets are composed of Ni, N, and C elements; and the Ni content on the surface is about 12 at%, similar to the theoretical value. After subtracting Shirley background, high‐resolution XPS spectra are fitted by mixture function of Lorentzian and Gaussian (**Figure** **3**a–c). The existence of Ni^ǁ^ can be testified in high‐resolution Ni 2p XPS spectrum (Figure 3a). The two typical peaks at 853.7 and 871.5 eV can be assigned to Ni 2p3/2 and Ni 2p1/2 of Ni–N_2_ moiety, while another two peaks at 859.9 and 877.5 eV can be assigned to the satellite peaks.^[^ ^37^, ^38^ ^]^ High‐resolution N 1s spectrum is deconvoluted into two peaks of C—N bond at 396.3 eV and Ni—N at 397.5 eV (Figure 3b).^[^ ^39^ ^]^ According to C spectrum, three subpeaks at 282.8, 283.9, and 286.6 eV can be assigned to the C 1s orbital of C=C, C—N, C—O, respectively (Figure 3c). All the analytic results indicate the Ni_3_(Ni_3_∙HAHATN)_2_ nanosheets are achieved in this work.

In theoretical, the conjugated ligand and Ni–N_4_ linkage can impart Ni_3_(Ni_3_∙HAHATN)_2_ with a fully π‐conjugated framework with excellent conductivity. Four‐point probe test manifests that the compressed pellet of Ni_3_(Ni_3_∙HAHATN)_2_ nanosheets possesses a high electrical conductivity with 2 S cm^−2^. Additionally, the density functional theory (DFT) calculation was carried out to estimate the electronic band structure of Ni_3_(Ni_3_∙HAHATN)_2_ by using the gradient‐corrected functional (GGA‐PBE) function (Figure 3d). The result of partial density of states (PDOS) indicates that single‐layered slab of Ni_3_(Ni_3_∙HAHATN)_2_ exhibits a very narrow band‐gap of ≈0.19 eV, suggesting that the Ni_3_(Ni_3_∙HAHATN)_2_ nanosheets should possess excellent conductivity for rapid electron transfer during the electrocatalytic process.

By regulating metal ionic species, a series of fully conjugated conductive MOFs M2_3_(M1_3_∙HAHATN)_2_ (M1:Co or Cu, M2:Ni or Cu) was obtained in this work (Figure S9, Supporting Information). The various products of M2_3_(M1_3_∙HAHATN)_2_ were confirmed by FTIR spectra (Figures S10 and S11, Supporting Information). After geometry optimization, all M2_3_(M1_3_∙HAHATN)_2_ exhibit 2D fully conjugated structures, similar to Ni_3_(Ni_3_∙HAHATN)_2_. All conductive MOFs belong to P6/mmm symmetry with analogous cell parameters. According to coordinated metal ionic species, M2_3_(M1_3_∙HAHATN)_2_ presents different M1–N_2_ moiety in in‐plane mesopores and M2–N_4_ linkage. DFT calculation indicates single‐layered M2_3_(M1_3_∙HAHATN)_2_ slabs all have very narrow band‐gap with neglectable differences (Figures S12–S14, Supporting Information). Owing to the analogous crystal structures, XRD patterns of three M2_3_(M1_3_∙HAHATN)_2_ exhibit obviously diffraction peaks in low angle (Figure S15, Supporting Information), similar to Ni_3_(Ni_3_∙HAHATN)_2_. TEM images show these M2_3_(M1_3_∙HAHATN)_2_ possess wrinkled thin‐layered morphologies with the thickness of 1–2 nm (Figure S16, Supporting Information). Meantime, homogeneous layered structures are also observed in SEM images of M2_3_(M1_3_∙HAHATN)_2_ (Figure S17, Supporting Information). More importantly, EDS mapping analysis shows two species of metal atoms uniformly distribute in the nanosheets of M2_3_(M1_3_∙HAHATN)_2_, which is direct evidence for the existence of two type metallic sites (M1–N_2_ and M2–N_4_) (Figures S18–S20, Supporting Information). The chemical composition and states of M2_3_(M1_3_∙HAHATN)_2_ were also determined by XPS measurements (Figures S21–S23, Supporting Information). Obviously, employing HAT structure as a conjugated ligand is an effective strategy to construct novel conductive MOFs with bimetallic sites, which is a significant experience for designing and synthesizing of next‐generation conductive MOFs with multiple functions.

**Figure 4:**
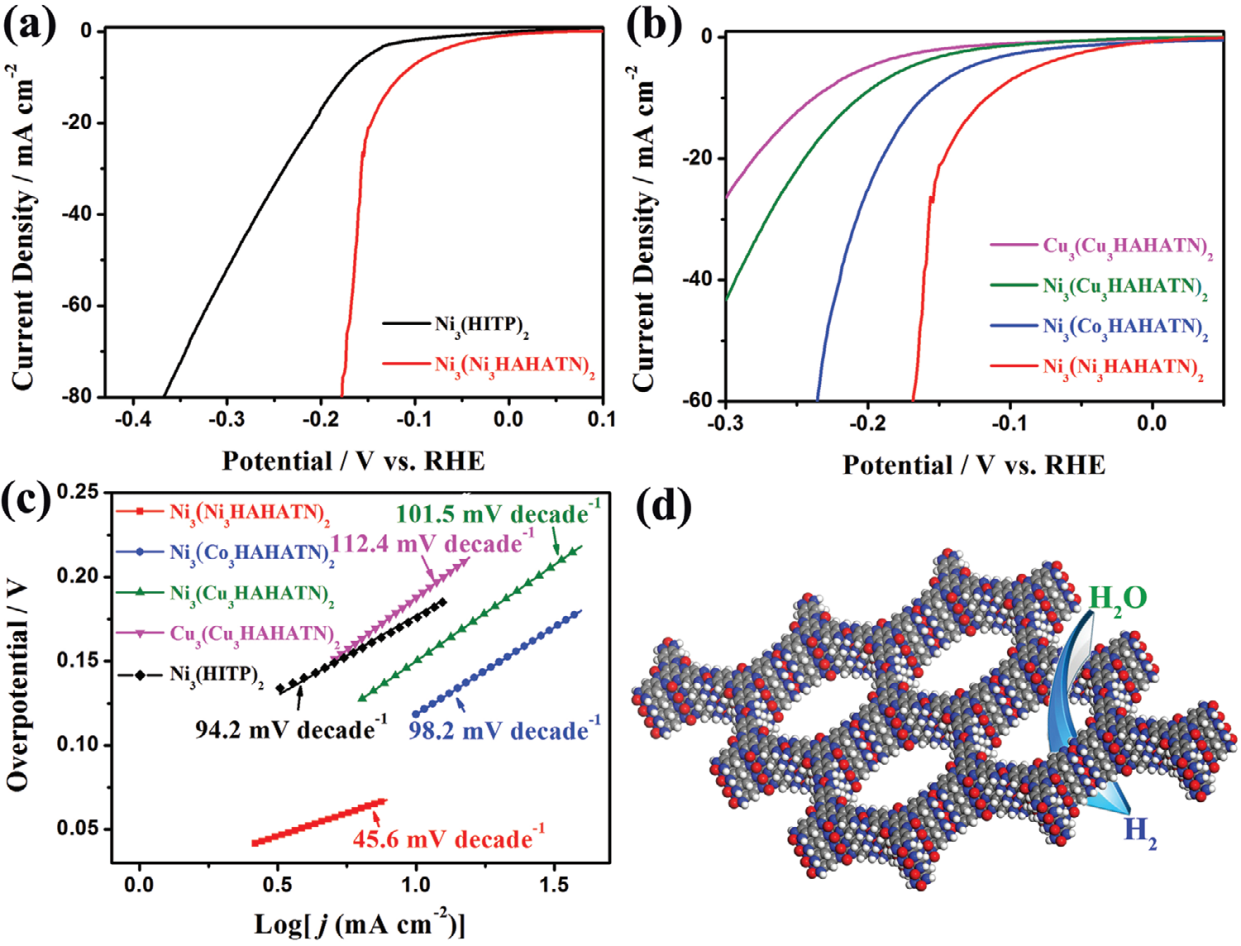
a) HER polarization curves of Ni_3_(HITP)_2_ and Ni_3_(Ni_3_∙HAHATN)_2_ samples. b) Polarization curves of the various M2_3_(M1_3_∙HAHATN)_2_ samples and c) the corresponding Tafel plots. d) Electrocatalytic diagram of Ni_3_(Ni_3_∙HAHATN)_2_ nanosheets toward HER.

The electrochemical property of Ni_3_(Ni_3_∙HAHATN)_2_ and traditional conductive MOFs Ni_3_(hexaiminotriphenylene)_2_ (Ni_3_(HITP)_2_) were investigated by various electrochemical technologies. In this work, all reported potentials were converted to reversible hydrogen electrode (RHE), and the current densities obtained were normalized by the geometric area of the working electrode. The linear sweep voltammetry (LSV) tests were carried out in N_2_‐saturated 0.1 m KOH solution at a scan rate of 5 mV s^−1^ to evaluate the HER activities of Ni_3_(Ni_3_∙HAHATN)_2_ and Ni_3_(HITP)_2_ (**Figure** **4**a). The LSV curves after iR correction (95%) display that Ni_3_(Ni_3_∙HAHATN)_2_ has an HER onset potential of 12 mV, which is much smaller than that (51 mV) of Ni_3_(HITP)_2_. The η_10_, defined as the overpotential at a current density of 10 mA cm^−2^, is 115 mV for Ni_3_(Ni_3_∙HAHATN)_2_, dramatically superior to the η_10_ of 176 mV for Ni_3_(HITP)_2_. The result explicitly indicates the Ni–N_2_ moiety plays a significant role in the HER activity of Ni_3_(Ni_3_∙HAHATN)_2_. Compared to the similar reports of MOF‐ or MOF‐derived electrocatalysts,^[^ ^15^, ^35^, ^40^, ^41^, ^42^, ^43^, ^44^, ^45^, ^46^, ^47^, ^48^, ^49^ ^]^ the electroactivity of Ni_3_(Ni_3_∙HAHATN)_2_ exhibits obviously superiority in this field (**Table** **1**). To investigate the effect of M1–N_2_ site, the HER activities of various Ni_3_(M1_3_∙HAHATN)_2_ samples were also evaluated by LSV measurement (Figure 4b). The η_10_ values of HER on Ni_3_(Cu_3_∙HAHATN)_2_, Ni_3_(Co_3_∙HAHATN)_2_, and Ni_3_(Ni_3_∙HAHATN)_2_ are 207, 162, and 115 mV, respectively. Obviously, the gradually enhanced HER activity indicates the active discrepancy of metal ionic species: Cu–N_2_ < Co–N_2_ < Ni–N_2_, which is another strong evidence for M1–N_2_‐based active center in M2_3_(M1_3_∙HAHATN)_2_. To further verify the active sites of M2_3_(M1_3_∙HAHATN)_2_, the electrocatalytic activity of Cu_3_(Cu_3_∙HAHATN)_2_ sample was tested. By comparison, the η_10_ value (230 mV) of Cu_3_(Cu_3_∙HAHATN)_2_ is just slightly larger than that of Ni_3_(Cu_3_∙HAHATN)_2_. Thus, this result reveals that the M1–N_2_ moiety plays a major role in the electroactivity of M2_3_(M1_3_∙HAHATN)_2_ toward HER. Additionally, cathodic current density is also an important factor to evaluate the HER activity of electrocatalyst. The Ni_3_(Ni_3_∙HAHATN)_2_ sample exhibits a high current density of 21.2 mA cm^−2^ at potentials of −0.15 V, about four times as high as that of Ni_3_(HITP)_2_. The HER current densities for Ni_3_(Co_3_∙HAHATN)_2_, Ni_3_(Cu_3_∙HAHATN)_2_, and Cu_3_(Cu_3_∙HAHATN)_2_ are 7.6, 3.2, and 1.9 mA cm^−2^, which are inferior to Ni_3_(Ni_3_∙HAHATN)_2_. Thus, the above experimental data further confirm the unsaturated M1–N_2_ site can serve as highly active center to remedy the shortcoming of traditional conductive MOFs toward electrocatalysis.

**Table 1:** Comparison of the HER activity for Ni_3_(Ni_3_∙HAHATN)_2_ with other MOF‐ or MOF‐derived electrocatalysts

Catalyst	Electrolyte	Overpotential^a)^ [mV]	Tafel plot [mV dec^−1^]	Ref.
Ni_3_(Ni_3_∙HAHATN)_2_	0.1 m KOH	115	45	This work
NiFe‐MOF/NF	1 m KOH	160	96	[40]
Ni‐ZIF@NF	1 m KOH	218	233	[41]
ZIF‐8 derived MoC	1 m KOH	182	60	[42]
MIL‐88A derived FeP	1 m KOH	95	72	[43]
HUST‐200@C	–	131	79	[44]
CoN_2_S_2_ MOFs	0.5 m H_2_SO_4_	283	71	[15]
Co‐BTSe	0.1 m KClO_4_	343	97	[45]
2D NiFe‐MOF/NF	1 m KOH	134	–	[35]
MOS 1	0.1 m KClO_4_	340	149	[46]
MOS 2	0.1 m KClO_4_	530	189	[46]
NENU‐500	0.5 m H_2_SO_4_	237	96	[47]
HKUST‐1	0.5 m H_2_SO_4_	691	127	[47]
GO/Cu‐MOF	0.5 m H_2_SO_4_	209^b)^	95	[48]

a) The overpotential at 10 mA cm^−2^

b) The overpotential at 30 mA cm^−2^.

Tafel slope is a key parameter to evaluate the HER kinetics. Tafel plots of M2_3_(M1_3_∙HAHATN)_2_ and Ni_3_(HITP)_2_ samples were obtained by the Tafel equation according to their LSV curves (Figure 4c). Tafel slope of Ni_3_(Ni_3_∙HAHATN)_2_ reaches up to 45.6 mV dec^−1^, which is far less than that of Ni_3_(HITP)_2_ (94.2 mV dec^−1^). Moreover, the Tafel slope values of Ni_3_(Co_3_∙HAHATN)_2_, Ni_3_(Cu_3_∙HAHATN)_2_, and Cu_3_(Cu_3_∙HAHATN)_2_ are 98.2, 101.5, and 112.4 mV dec^−1^, respectively. Among these conductive MOFs, Ni_3_(Ni_3_∙HAHATN)_2_ exhibits the smallest Tafel slope, indicating the fastest kinetics toward HER electrocatalysis.

**Figure 5:**
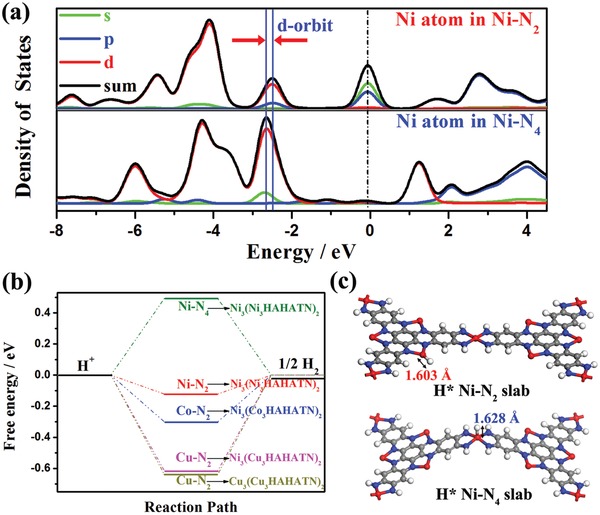
a) PDOS of Ni atom in Ni–N_2_ and Ni–N_4_ of Ni_3_(Ni_3_∙HAHATN)_2_ slab. b) Free‐energy profiles toward HER at various electrocatalytic sites. c) The hydrogen adsorption slabs of Ni_3_(Ni_3_∙HAHATN)_2_ at Ni–N_2_ and Ni–N_4_ sites.

In general, the electrocatalytic activity relates to the conductivity of catalysts for effective electron transfer. PDOS calculation and four‐probe measurement demonstrate the rigid conjugated structure endows M2_3_(M1_3_∙HAHATN)_2_ with an excellent electrical conductivity, which is beneficial for electron transfer during HER. Moreover, the SEM image exhibits the Ni_3_(Ni_3_∙HAHATN)_2_ membrane on electrode still maintain layered structure with hierarchical porosity after fabrication process of the modified electrode (Figure S24, Supporting Information). Benefiting from the advantage of the unique porous morphology, the Ni_3_(Ni_3_∙HAHATN)_2_ facilitate mass transfer during the electrocatalytic process (Figure 4d). Theoretically, only an unsaturated site can provide binding affinity to proton to achieve electron transfer. In this work, the M1–N_2_ moiety of M2_3_(M1_3_∙HAHATN)_2_ has a higher unsaturation degree in comparison with that of M–N_4_ linkage. For instance, the PDOS results show the electronic orbits (s and p‐orbits) of Ni, coordinated by bidentate tertamine (Ni–N_2_), are located beside the Fermi level in Ni_3_(Ni_3_∙HAHATN)_2_ slab, but the location of electronic orbits of Ni in linkages (Ni–N_4_) is far away (**Figure** **5**a). Moreover, the d‐orbit of Ni atoms in Ni–N_2_ moieties is also closer to Fimi level than that of the Ni atoms in Ni–N_4_ linkages, which indicates the Ni–N_2_ moieties have stronger absorption and bonding capacity for proton.^[^ ^50^ ^]^ Similar phenomenon occurs on the other M2_3_(M1_3_∙HAHATN)_2_ slabs as well (Figures S25 and S26, Supporting Information). To further investigate the active site of M2_3_(M1_3_∙HAHATN)_2_, the free energies of hydrogen adsorption (Δ*G*_H*_) were calculated via DFT. According to thermodynamics and kinetics, the optimal Δ*G*_H*_ should be approached to thermoneutral 0 eV.^[^ ^51^ ^]^ The DFT simulation was also performed to calculate the values of Δ*G*_H*_ of various M2_3_(M1_3_∙HAHATN)_2_ slabs (Figure 5b). In Ni_3_(Ni_3_∙HAHATN)_2_, the Ni site of Ni–N_4_ linkage expresses an exothermic Δ*G*_H*_ of +0.49 eV, which indicates that the Ni–N_4_ linkage is hard to combine with protons. The adsorption energy of H* of Ni atom in Ni–N_2_ moiety decreases to −0.12 eV, which is close to the optimal zero overpotential (Δ*G*_H_ _*_ = 0 eV). This phenomenon illustrates the Ni–N_2_ site need lesser energy to break the bond of Ni‐H to complete the catalytic process for HER. Similarly, the length of N—H bond in Ni–N_2_ site is 1.603 Å, smaller than that in Ni–N_4_ linkage (1.628 Å), suggesting the proton is easier to adsorb on the Ni site of Ni–N_2_ moiety (Figure 5c). And hydrogen absorption in Ni–N_4_ linkage makes the framework of Ni_3_(Ni_3_∙HAHATN)_2_ bending, which breaks the rigid 2D structure. These results show the Ni–N_2_ moieties in Ni_3_(Ni_3_∙HAHATN)_2_ is more active for HER than Ni–N_4_ linkages in conductive MOFs. Meantime, the Δ*G*_H*_ of M1–N_2_ center in M2_3_(M1_3_∙HAHATN)_2_ slabs also exhibits a similar regularity as the electrochemical results: Ni–N_2_ in Ni_3_(Ni_3_∙HAHATN)_2_ (−0.12 eV) > Co–N_2_ in Ni_3_(Co_3_∙HAHATN)_2_ (−0.29 eV) > Cu–N_2_ in Ni_3_(Cu_3_∙HAHATN)_2_ (−0.61 eV) > Cu–N_2_ in Cu_3_(Cu_3_∙HAHATN)_2_ (−0.64 eV). According to these results, M2_3_(M1_3_∙HAHATN)_2_ is expected to be an ideal coordinated structure toward electrocatalysis, which proves our design concept of new conductive MOFs for electrocatalysis is feasible.

**Figure 6:**
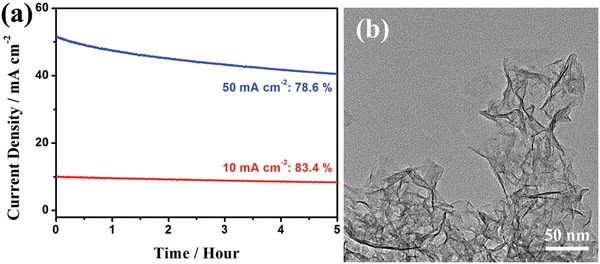
a) Time‐dependent HER current density curves for Ni_3_(Ni_3_∙HAHATN)_2_ sample at 10 and 50 mA cm^−2^. b) TEM image of Ni_3_(Ni_3_∙HAHATN)_2_ sample after chronoamperometry test.

The durability is another parameter to evaluate the practicability of electrocatalysts. The HER stability of Ni_3_(Ni_3_∙HAHATN)_2_ was primarily investigated by repeating CV tests in a 0.1 m KOH solution. After 1000 cycles, the overpotential of Ni_3_(Ni_3_∙HAHATN)_2_ only displays a tiny deformation, relative to that of the initial scan (Figure S27, Supporting Information). The result indicates the Ni_3_(Ni_3_∙HAHATN)_2_ sample possesses promising electrocatalytic stability for HER. Sequentially, the excellent durability of Ni_3_(Ni_3_∙HAHATN)_2_ was further confirmed by chronoamperometry measurement at 10 mA cm^−2^. After 10 h test, the HER current reveals a negligible attenuation, which maintains 83.4% of initial activity (**Figure** **6**a). Meantime, the attenuation of Ni_3_(Ni_3_∙HAHATN)_2_ still keep at a low level (78.6%) at a high current density of 50 mA cm^−2^. On the other hand, the XRD pattern of Ni_3_(Ni_3_∙HAHATN)_2_ maintains the particular crystal structure after electrochemical test (Figure S28a, Supporting Information). Similarly, the TEM and SEM characterizations show the electrocatalytic process cannot break the 2D structure of Ni_3_(Ni_3_∙HAHATN)_2_ nanosheets (Figure 6b and Figure S28b, Supporting Information). The results reveal that the rigid conjugated coordinated structure makes Ni_3_(Ni_3_∙HAHATN)_2_ possess excellent durability during HER process.

## 3 Conclusion

To better exert the structural advantages of conductive MOFs, a type of new conductive M2_3_(M1_3_∙HAHATN)_2_ MOFs with extra metallic sites (M1–N_2_) was designed and prepared by employing a novel conjugated ligand. The larger ligand molecule endows M2_3_(M1_3_∙HAHATN)_2_ with an expanded in‐plane porous structure (2.7 nm) for mass transfer. DFT calculations and four‐probe measurement clearly demonstrate the M2_3_(M1_3_∙HAHATN)_2_ nanosheets possess excellent conductive ability for electron transfer. Importantly, the introduced metal atom in M1–N_2_ moiety exhibits a higher unsaturated degree than that in traditional M2–N_4_ linkage. The DFT calculations (PDOS and free energies of hydrogen adsorption) predict the major active center for HER in M2_3_(M1_3_∙HAHATN)_2_ is M1–N_2_ moiety rather than M2–N_4_ linkage. Subsequently, a series of HER electrocatalytic tests confirm the prophecy of theory in experimental. The optimal Ni_3_(Ni_3_∙HAHATN)_2_ nanosheets exhibit a remarkably activity enhancement for HER compared to traditional Ni_3_(HITP)_2_ conductive MOF. The rigid layered structure imparts Ni_3_(Ni_3_∙HAHATN)_2_ nanosheets with an excellent durability. These characteristics make Ni_3_(Ni_3_∙HAHATN)_2_ as a promising electrocatalyst for HER, and confirm our design concept of new conductive MOFs is meaningful for electrocatalysis.

## 4 Experimental Section

##### Reagents and Chemicals

1,2,4,5‐benzenetetramine tetrahydrochloride (Scheme S4a, Supporting Information), and hexaketocyclohexane octahydrate (Scheme S4b, Supporting Information) were purchased from Sigma‐Aldrich, USA. HITP ligand was obtained from TCI (Shanghai), China. Nickel(II) chloride, cobalt(II) chloride, cupric(II) chloride, and ammonium hydroxide were obtained from Macklin reagent company, China. Ether, ethanol and *N*,*N*‐dimethylformamide (DMF) were purchased from Beijing Chemical Reagent Company, China. All commercial chemicals were directly used without further purification.

##### Preparation of HAHATN

HAHATN was prepared by a microwave synthetic strategy, according to the procedure available in previous works.^[^ ^48^ ^]^ Briefly, 702 mg of 1,2,4,5‐benzenetetramine tetrahydrochloride (2.47 mmol) and 224 mg of hexaketocyclohexane octahydrate (0.718 mmol) were dispersed in 10 mL ethanol with concentrated hydrochloric acid (38%, 2 mL). The suspension was transferred to a microwave reaction vessel and reacted at 160 °C for 45 min. Then, the resulting solution was filtered to remove black byproduct and further concentrated under vacuum to dryness. The black HAHATN was obtained by recrystallizing in ethanol and washed twice with ether (225 mg, 45% yield).

##### Preparation of Ni_3_∙HAHATN Ligand

Ni_3_∙HAHATN ligand was prepared by a selective coordinated process. 162 mg of nickel chloride was dissolved in 30 mL ethanol, and the salt solution was adjusted pH by hydrochloric acid to 4. Then 100 mg of HAHATN was added into the acidic salt solution and refluxed for 4 h. The black Ni_3_∙HAHATN ligand was obtained by recrystallizing in ethanol to remove unreacted nickel chloride.

##### Preparation of Co_3_∙HAHATN Ligand

Co_3_∙HAHATN ligand was prepared by a similar method. In this process, 162 mg of nickel chloride was replaced by 159 mg of cobalt chloride to coordinate with HAHATN. The yield of Co_3_∙HAHATN ligand was 102 mg.

##### Preparation of Cu_3_∙HAHATN Ligand

Cu_3_∙HAHATN ligand was also prepared by a similar method. 162 mg of nickel chloride was replaced by 164 mg of cupric chloride to coordinate with HAHATN. The yield of Co_3_∙HAHATN ligand was 95 mg.

##### Preparation of Ni_3_(M1_3_∙HAHATN)_2_ Conductive MOFs

Ni_3_(M1_3_∙HAHATN)_2_ was obtained by a coordinated reaction in alkaline. 51 mg of nickel chloride was dissolved in 42 mL deionized water with ammonium hydroxide (2.5 mL), and the mixed solution was stirred at 65 °C under air atmosphere. Then, 100 mg of M1_3_∙HAHATN ligand was added into the solution with continuous stirring for 2 h. The product was purified by washing with ethanol and deionized water.

##### Preparation of Cu_3_(Cu_3_∙HAHATN)_2_ Conductive MOFs

Cu_3_(Cu_3_∙HAHATN)_2_ was obtained by a similar method, 51 mg of nickel chloride was replaced by 53 mg of cupric chloride to coordinate with Cu_3_∙HAHATN ligand.

##### Preparation of Ni_3_(HITP)_2_ Conductive MOFs

Ni_3_(HITP)_2_ conductive MOFs was synthesized according to the previous report.^[^ ^26^ ^]^ 6.6 mg of nickel chloride was dissolved in 5 mL of water and 0.3 mL of concentrated aqueous ammonia. 10 mg of HITP solution (5 mL of water) was added into the above‐mentioned salt solution. This mixture was stirred at 65 °C for 3 h. The resulting black powder was centrifuged, filtered, and then washed by ethanol and deionized water. Then, the solid was dried under vacuum at 150 °C.

##### Characterization

NMR spectra were obtained by 400 MHz Bruker ASCEND NMR Spectrometer. D/Max‐3cX′Pert was employed to record the XRD patterns. The SEM images and EDX mapping were taken by a HITACHI SU8020 field‐emission electron microscope. TEM images were made on a TECNAI F20 transmission electron microscope. PHI‐5000 X‐ray photoelectron spectrometer was used for XPS measurement. Nitrogen sorption experiments were performed with a nitrogen physical adsorption instrument (ASAP 2400). Before testing, Ni_3_(Ni_3_∙HAHATN)_2_ sample was firstly degassed at 150 °C for 10 h.

##### Preparation of Electrode

In this work, rotating disk glassy carbon electrode (RDE) with 3 mm in diameter was employed as the working electrode. Before preparing the modified electrode, the working electrode was firstly treated by pre‐polishing to remove contaminant on the surface of the electrode. On the other side, 8 mg of various M2_3_(M1_3_∙HAHATN)_2_ samples were dispersed in 2 mL mixed solvent (*V*_water_:*V*_DMF_ = 1:1) and ultrasonicated for 10 min to achieve a uniform ink. 10 µL of well‐dispersed sample ink was dropped onto the pre‐treated RDE and dried in vacuum. Then, 5 µL of Nafion ethanol solution was used to prevent electrocatalyst from falling off.

##### Electrochemical Measurement

The HER electrochemical measurements were performed on in CHI 760E electrochemical workstation with a three‐electrode system. The counter electrode and reference electrode were employed by graphite and saturated calomel electrode, respectively. The electrochemical measurements were carried out in N_2_‐saturated 0.1 m KOH electrolyte and all potentials in this work were transformed to RHE via the following equation

(1) $$E_{\text{RHE}}=E_{SCE}+0.243\;\text{V}+0.059\times \text{pH}$$

LSV measurements were performed at 1600 rpm in 5 mV s^−1^ with 95% iR corrections to evaluate their activities.

##### DFT Calculation

DFT simulation of various M2_3_(M1_3_∙HAHATN)_2_ slabs were calculated by CASTEP and DMol^3^ modules. In these slabs, a vacuum layer of 30 Å was established to avoid periodic interaction. And, PDOS calculation was performed by GGA+U functional with additional Coulomb potential (*U*_Ni_ = 3.1, *U*_Co_ = 3.1 and *U*_Cu_ = 3.0 eV) for 3d‐orbit. A plane‐wave energy cut off of 400 eV was used together with norm‐conserving pseudopotentials, and the Brillouin zone was sampled with a 2 × 2 × 1 Monkhorst‐Pack grid. The structure was fully optimized until the force on each atom was less than 10^−3^ eV Å^−1^. On the other side, the free energy (∆*G*) was computed from

(2) ΔG=ΔE+ZPE−TΔS

where ∆*E* means the total energy, ZPE was the zero‐point energy, the entropy (∆*S*) of each adsorbed state were obtained from DFT calculation, whereas the thermodynamic corrections for gas molecules were from standard tables.

## Conflict of Interest

The authors declare no conflict of interest.

## Supporting information

Supporting Information
