# Supplementary material for: Conductive Metal–Organic Frameworks with Extra Metallic Sites as an Efficient Electrocatalyst for the Hydrogen Evolution Reaction
Source: Adv Sci (Weinh). 2020 Mar 16;7(9):2000012. doi: 10.1002/advs.202000012 (PMC7201256; doi:10.1002/advs.202000012)
Supplement: Supplementary file 1 — Supporting Information [file ADVS-7-2000012-s001.pdf]

Copyright WILEY-VCH Verlag GmbH & Co. KGaA, 69469 Weinheim, Germany, 2020.

ADVANCED SCIENCE
Open Access

Supporting Information

for *Adv. Sci.,* DOI: 10.1002/advs.202000012

Conductive Metal–Organic Frameworks with Extra Metallic Sites as an Efficient Electrocatalyst for the Hydrogen Evolution Reaction

Hao Huang, Yue Zhao, Yimin Bai, Fumin Li, Ying Zhang, and Yu Chen**

Copyright WILEY-VCH Verlag GmbH & Co. KGaA, 69469 Weinheim, Germany, 2020.

Supporting Information

Conductive Metal-Organic Frameworks with Extra Metallic Sites as Efficient Electrocatalyst for Hydrogen Evolution Reaction

*Hao Huang[a], Yue Zhao[b], Yimin Bai[a], Fumin Li[a], Ying Zhang[*a] and Yu Chen[*b]*

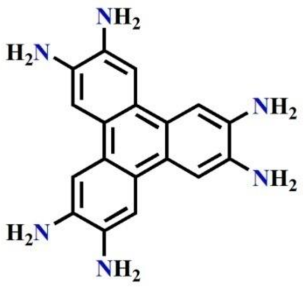

Scheme S1. Chemical structure of hexaiminotriphenylene (HITP).

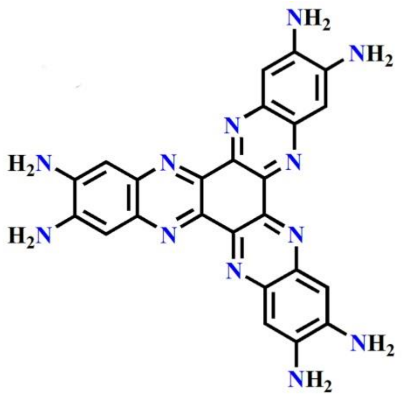

Scheme S2. Chemical structure of hexaiminohexaazatrinaphthalene (HAHATN).

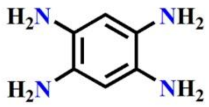

Scheme S3. Chemical structure of 1,2,4,5-benzenetetramine tetrahydrochloride.

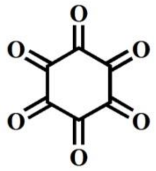

Scheme S4. Chemical structure of hexaketocyclohexane octahydrate.

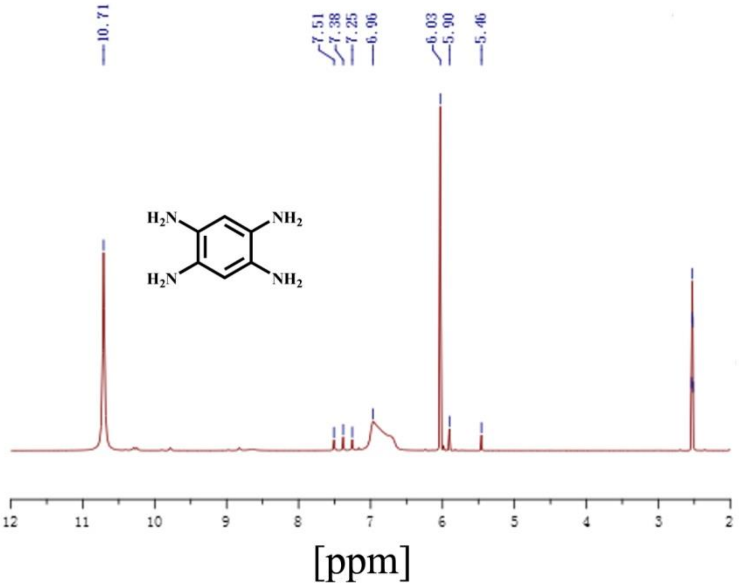

Figure S1. ^{1}H HMR of 1,2,4,5-benzenetetramine tetrahydrochloride in dry DMSO-d_6.

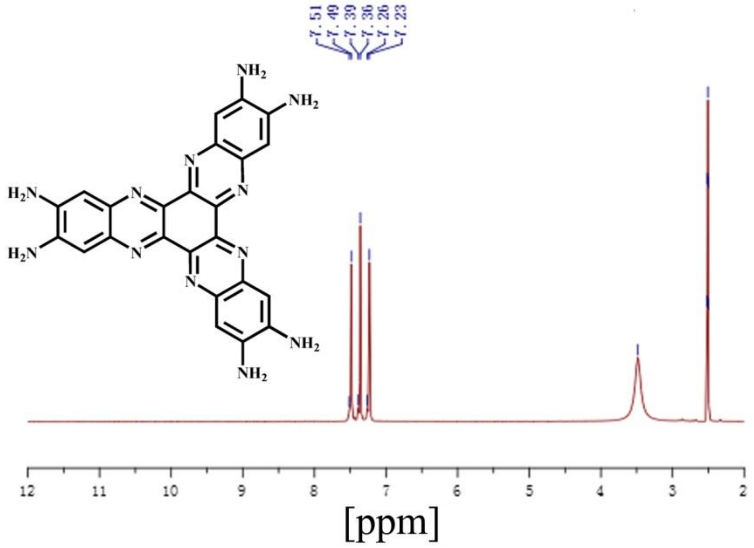

Figure S2. ^{1}H HMR of HAHATN in dry DMSO-d_6.

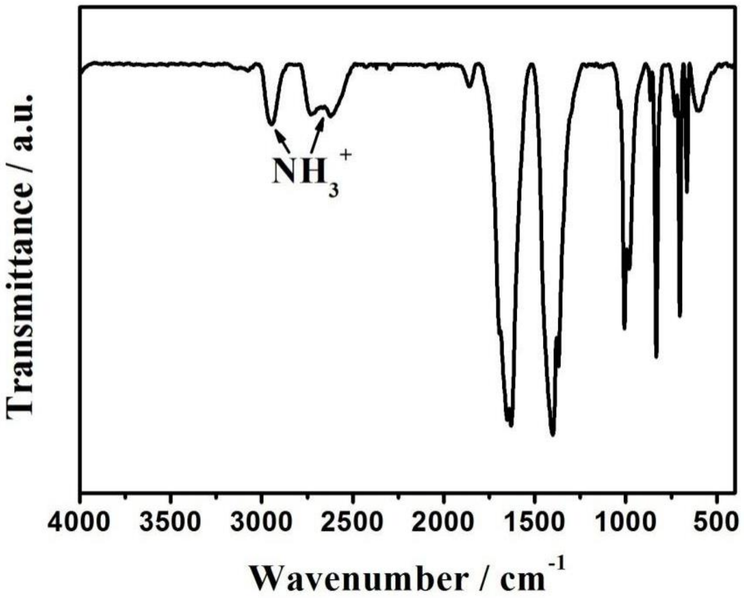

Figure S3. FT-IR spectrum of HAHATN.

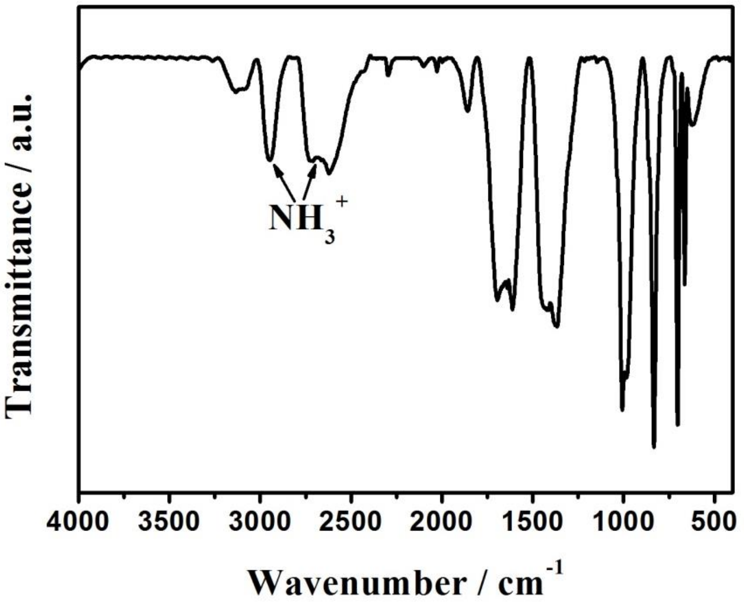

Figure S4. FT-IR spectrum of Ni_3·HAHATN ligand.

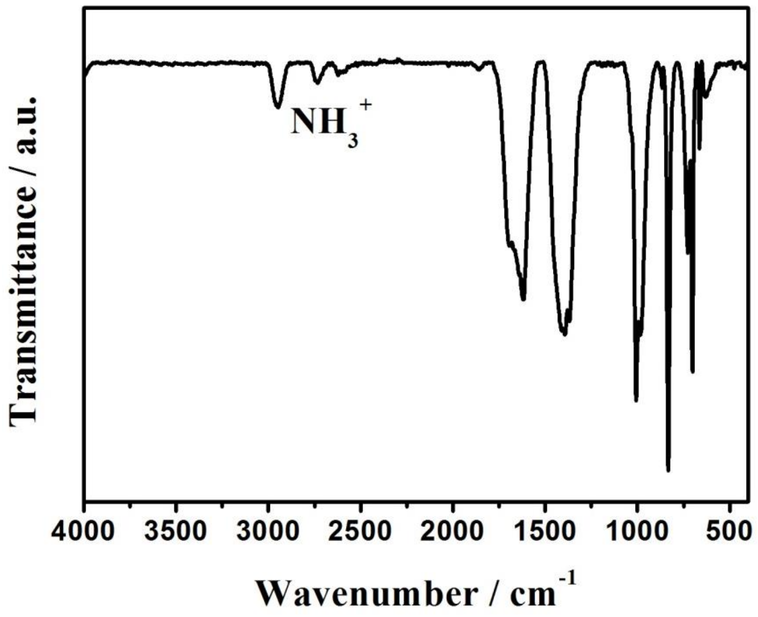

Figure S5. FT-IR spectrum of $Ni_3(Ni_3{\cdot}HAHATN)_2$.

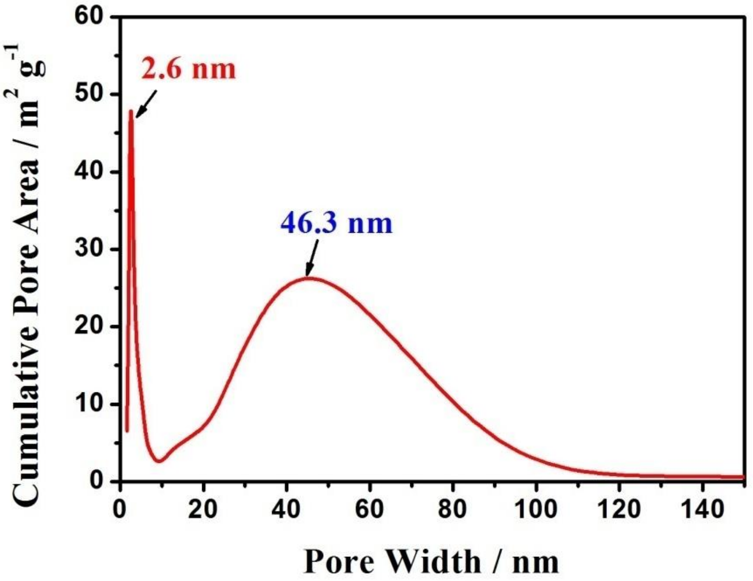

Figure S6. Pore size distribution curve of $Ni_3(Ni_3{\cdot}HAHATN)_2$ nanosheets.

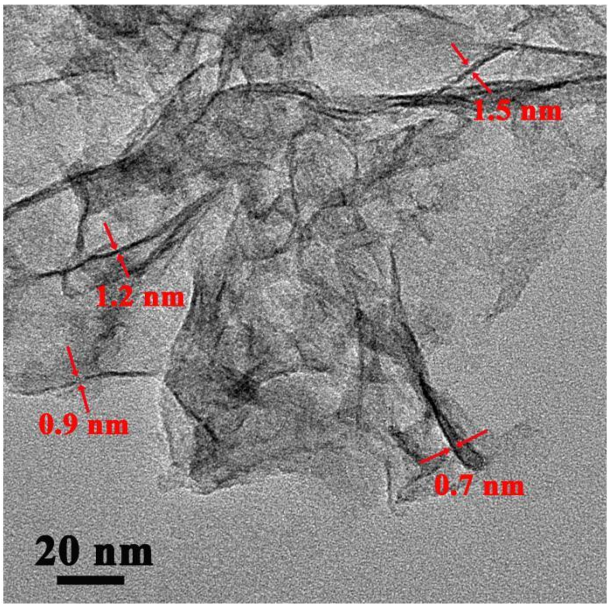

Figure S7. HRTEM image of $Ni_3(Ni_3{\cdot}HAHATN)_2$ nanosheets.

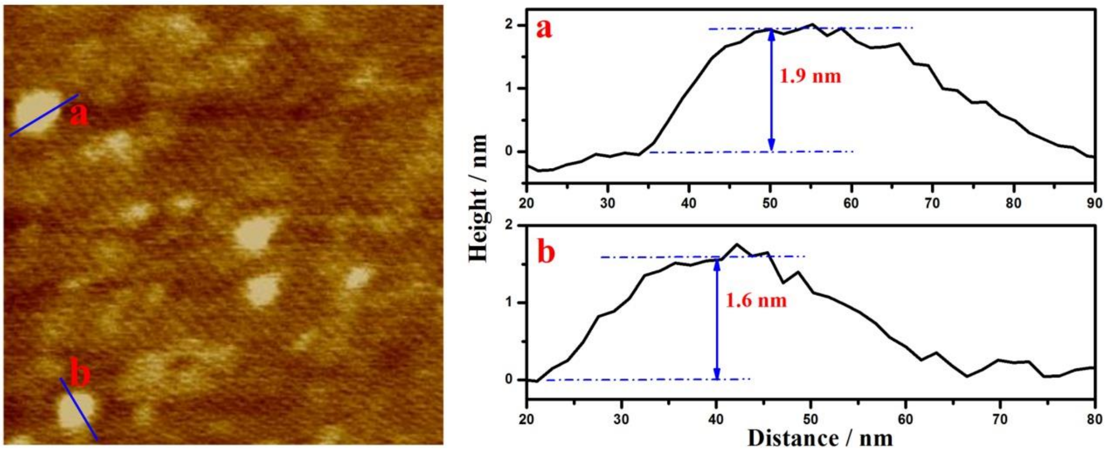

Figure S8. AFM image and height profile of $Ni_3(Ni_3{\cdot}HAHATN)_2$ nanosheets.

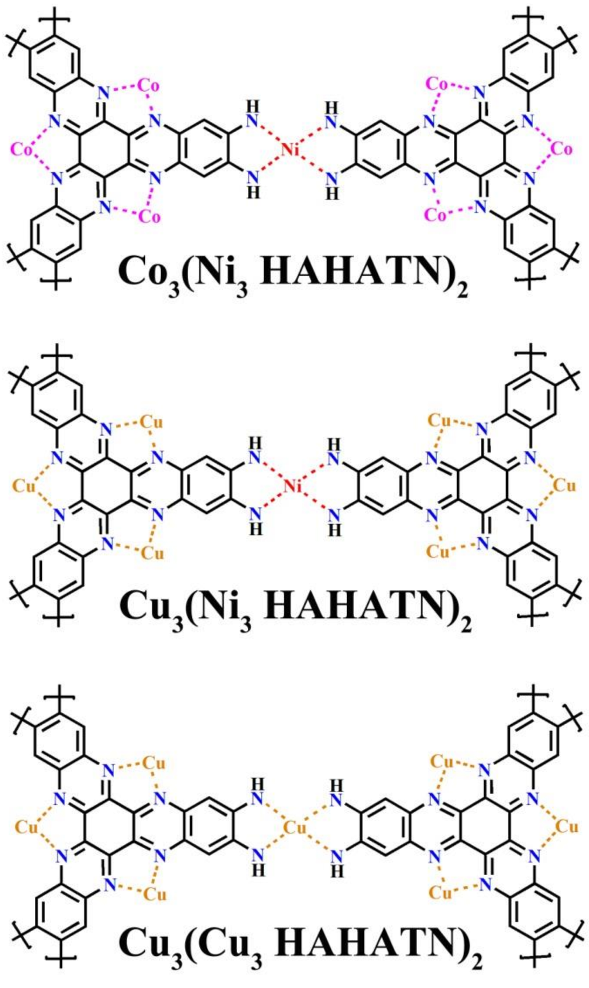

Figure S9. Chemical structure of various $M2_3(M1_3{\cdot}HAHATN)_2$.

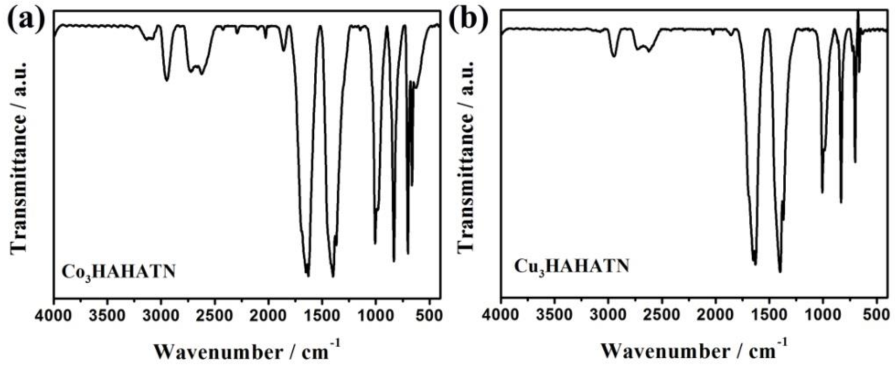

Figure S10. FT-IR spectra of (a) Co_3·HAHATN and (b) Cu_3·HAHATN ligand.

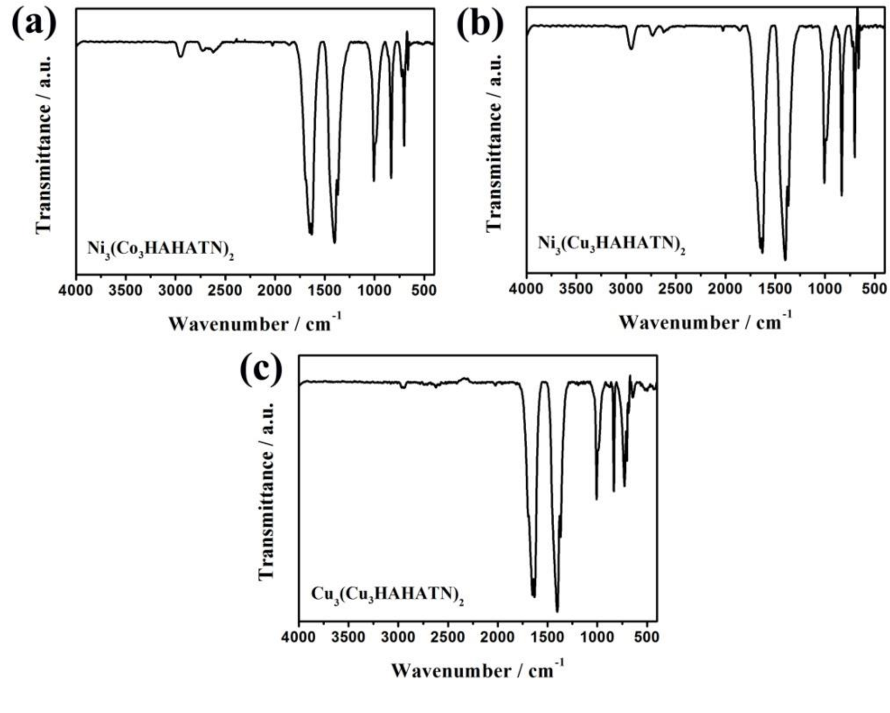

Figure S11. FT-IR spectra of (a) $Ni_3(Co_3{\cdot}HAHATN)_2$, (b) $Ni_3(Cu_3{\cdot}HAHATN)_2$, (c) $Cu_3(Cu_3{\cdot}HAHATN)_2$ nanosheets.

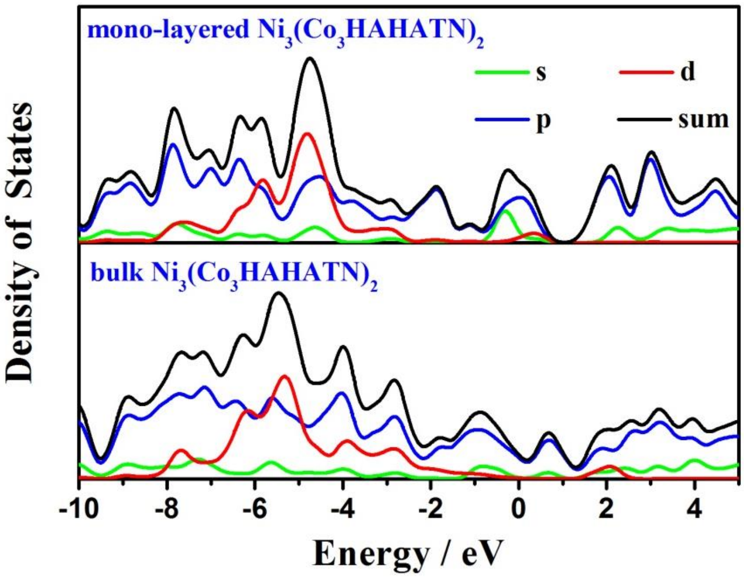

Figure S12. Calculated partial density of states (PDOS) of mono-layered and bulk $Ni_3(Co_3{\cdot}HAHATN)_2$ slabs.

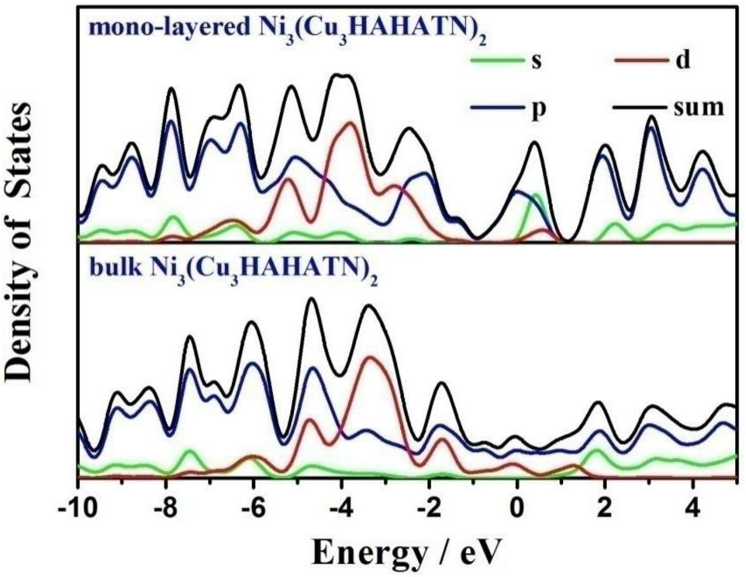

Figure S13. Calculated PDOS of mono-layered and bulk $Ni_3(Cu_3{\cdot}HAHATN)_2$ slabs.

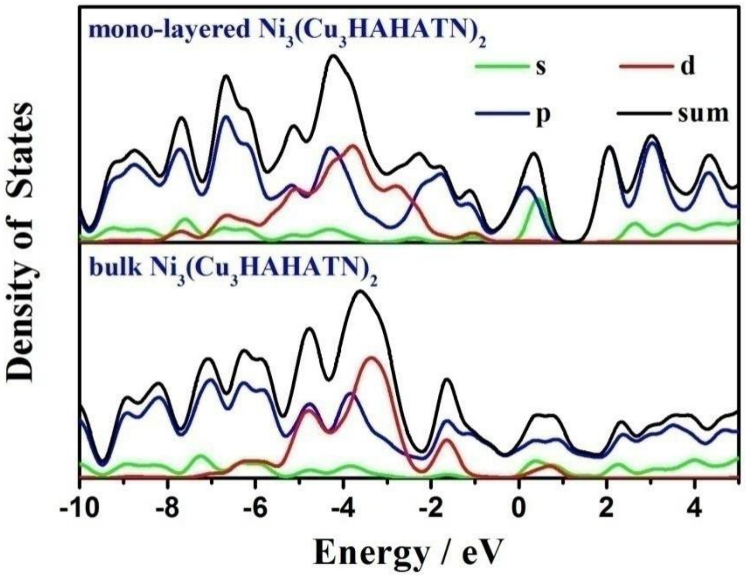

Figure S14. Calculated PDOS of mono-layered and bulk $Cu_3(Cu_3{\cdot}HAHATN)_2$ slabs.

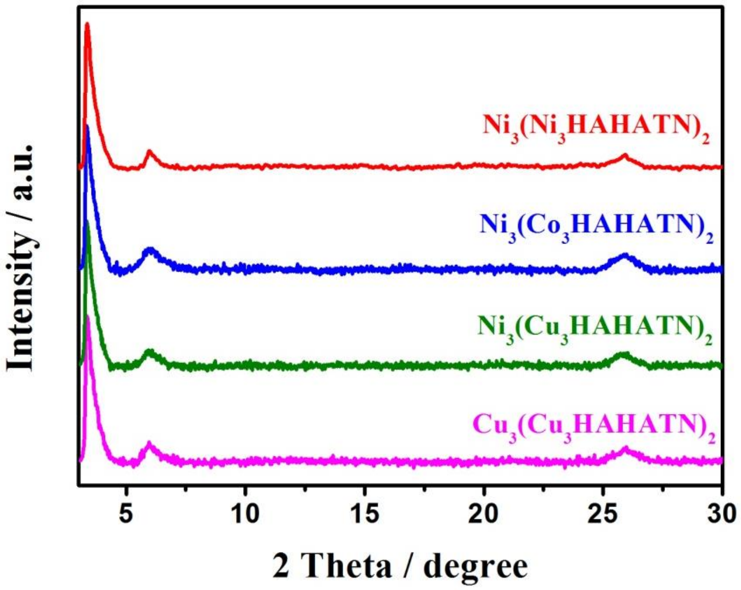

Figure S15. XRD patterns of $Ni_3(Co_3{\cdot}HAHATN)_2$, $Ni_3(Cu_3{\cdot}HAHATN)_2$ and $Cu_3(Cu_3{\cdot}HAHATN)_2$ samples.

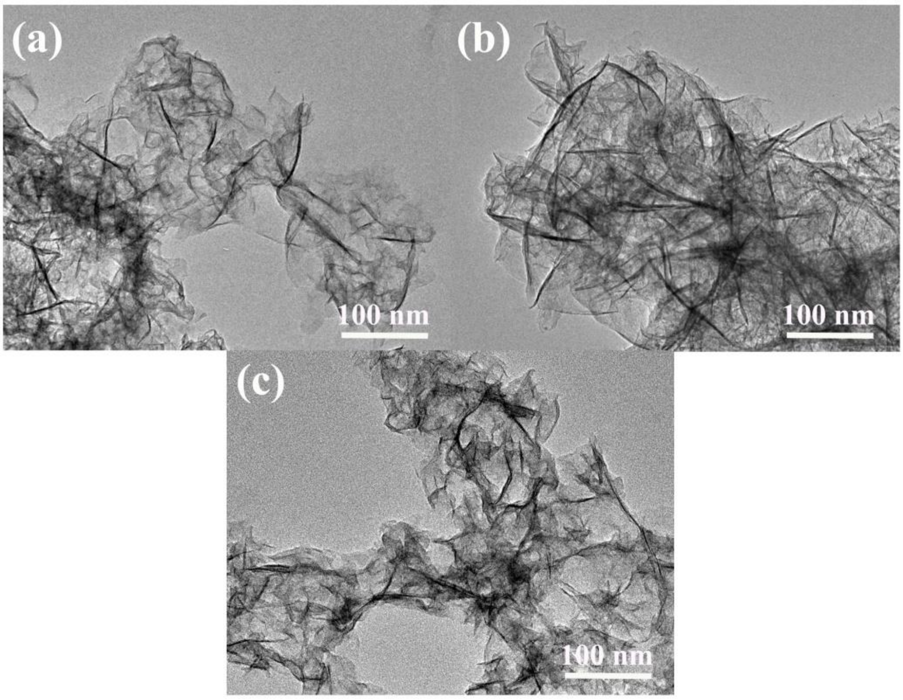

Figure S16. TEM images of (a) $Ni_3(Co_3{\cdot}HAHATN)_2$, (b) $Ni_3(Cu_3{\cdot}HAHATN)_2$, (c) $Cu_3(Cu_3{\cdot}HAHATN)_2$ samples.

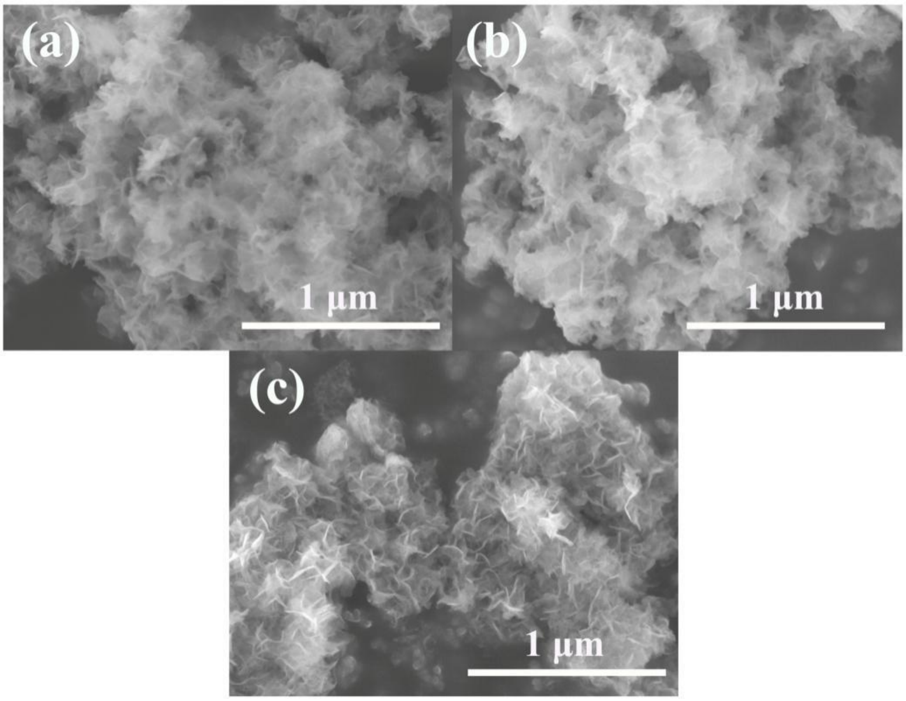

Figure S17. SEM images of (a) $Ni_3(Co_3{\cdot}HAHATN)_2$, (b) $Ni_3(Cu_3{\cdot}HAHATN)_2$, (c) $Cu_3(Cu_3{\cdot}HAHATN)_2$ samples.

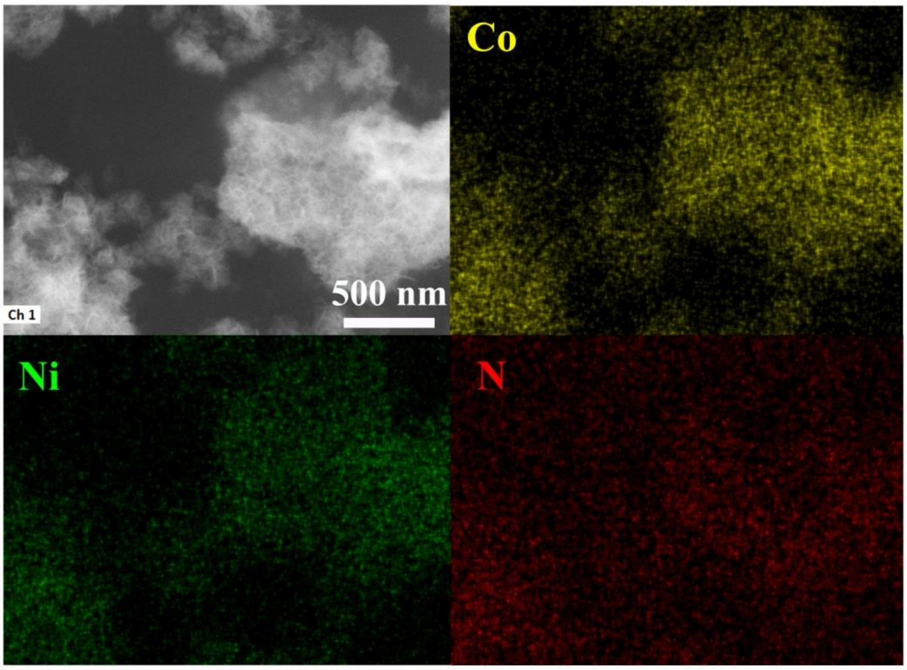

Figure S18. EDX mapping of $Ni_3(Co_3 \cdot HAHATN)_2$ sample.

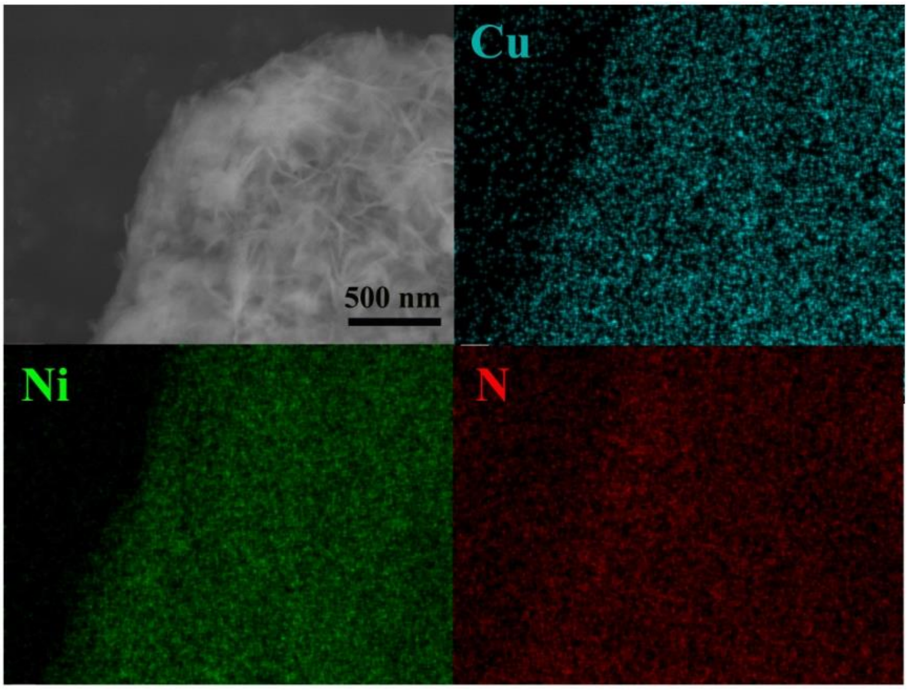

Figure S19. EDX mapping of $Ni_3(Cu_3 \cdot HAHATN)_2$ sample.

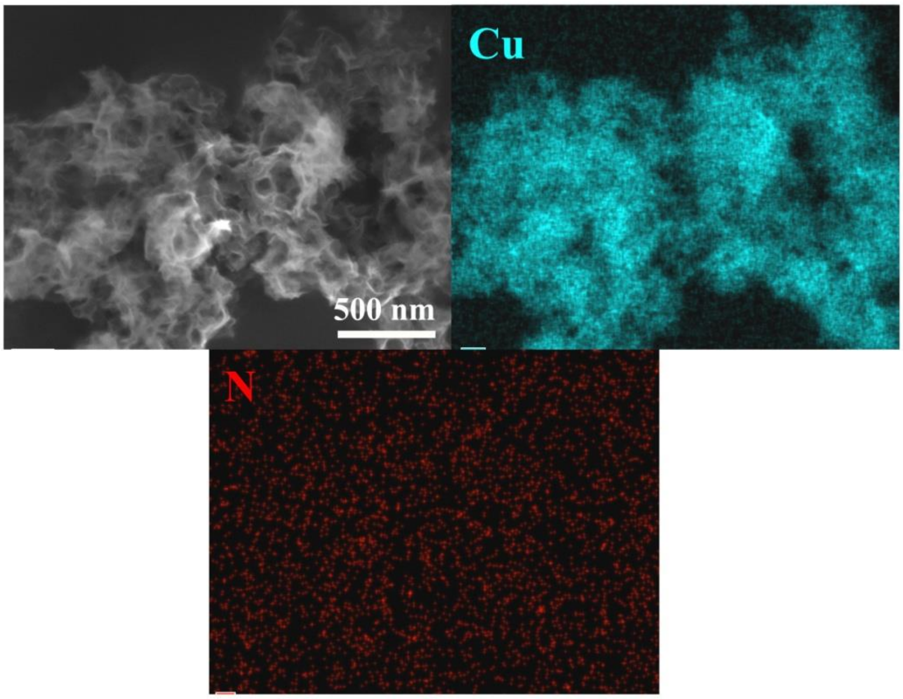

Figure S20. EDX mapping of $Cu_3(Cu_3{\cdot}HAHATN)_2$ sample.

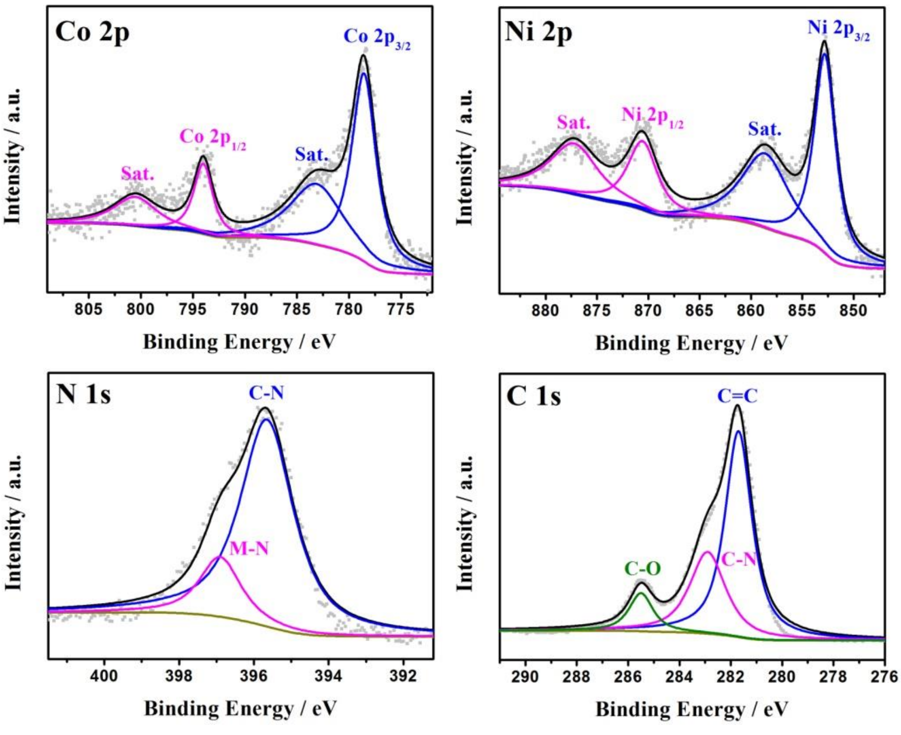

Figure S21. XPS spectra of $Ni_3(Co_3{\cdot}HAHATN)_2$ sample.

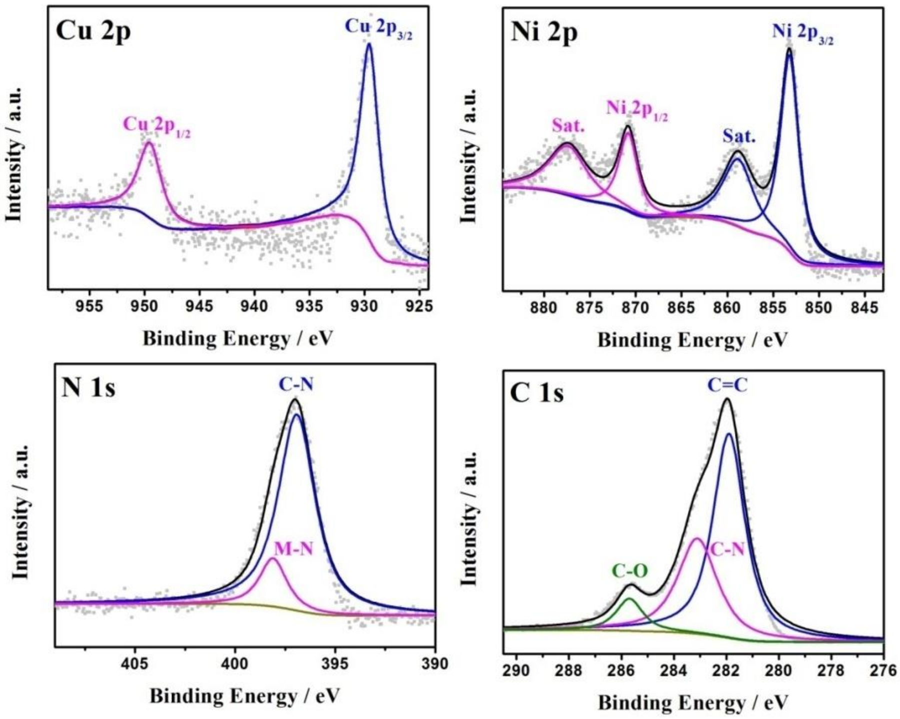

Figure S22. XPS spectra of $Ni_3(Cu_3{\cdot}HAHATN)_2$ sample.

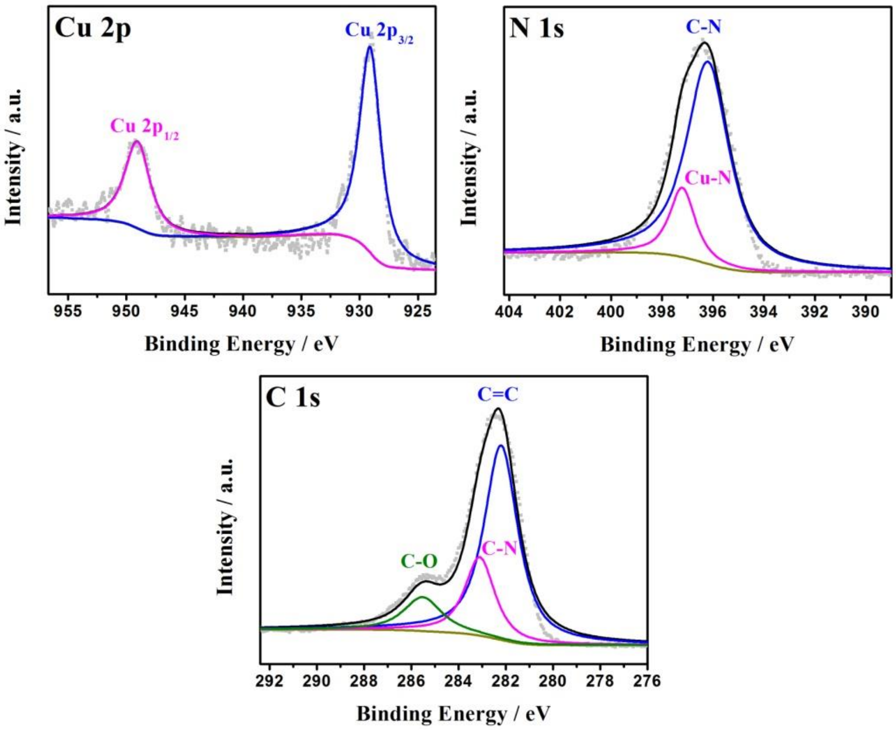

Figure S23. XPS spectra of $Cu_3(Cu_3{\cdot}HAHATN)_2$ sample.

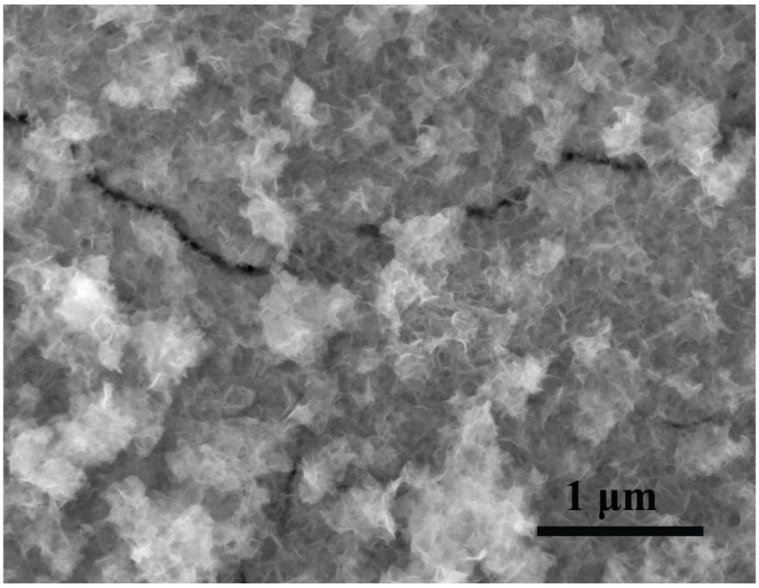

Figure S24. SEM image of $Ni_3(Ni_3{\cdot}HAHATN)_2$ membrane on electrode.

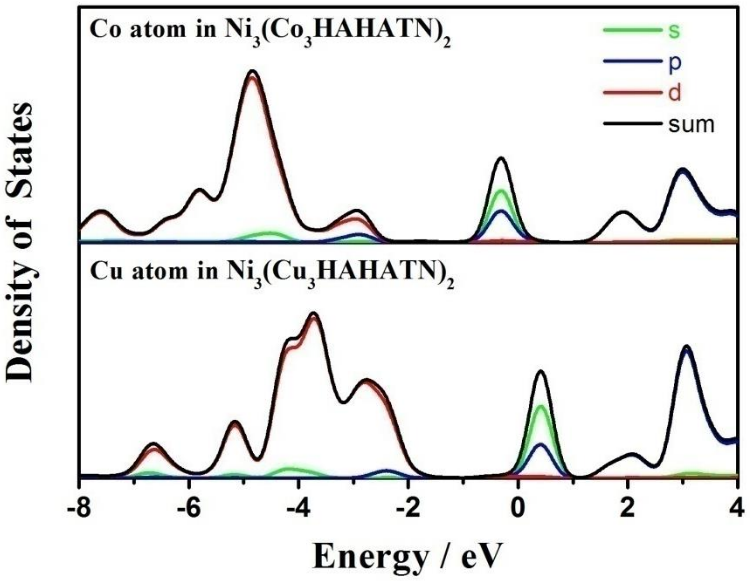

Figure S25. Calculated PDOS of Co atom in Co-N_2 of $Ni_3(Co_3{\cdot}HAHATN)_2$ and Cu atom in Cu-N_2 of $Ni_3(Cu_3{\cdot}HAHATN)_2$ slabs.

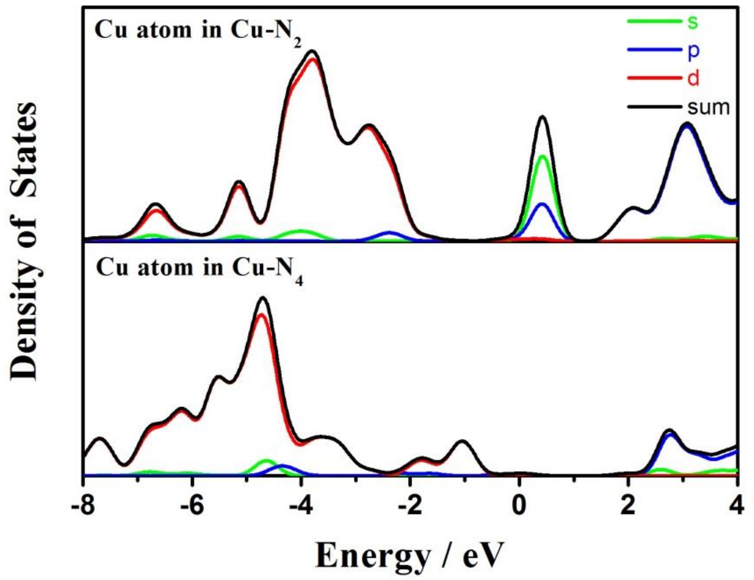

Figure S26. Calculated PDOS of Cu atom in Cu-N_2 and Cu-N_4 of $Cu_3(Cu_3{\cdot}HAHATN)_2$ slab.

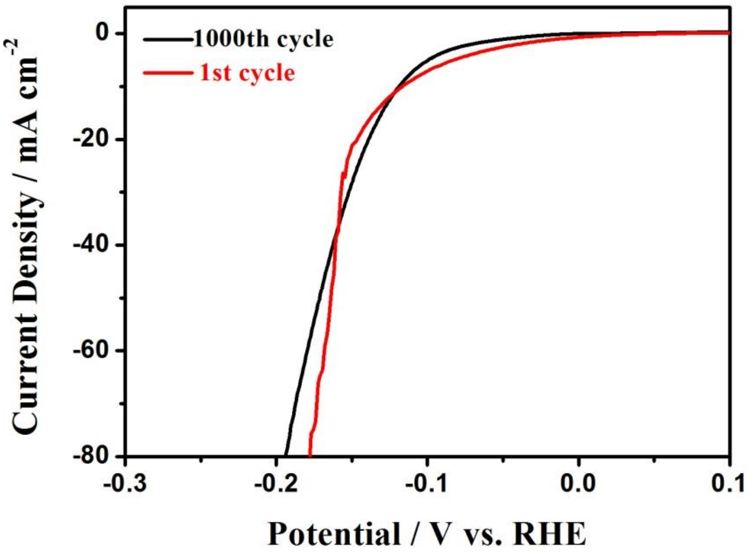

Figure S27. LSV polarization curves for $Ni_3(Ni_3{\cdot}HAHATN)_2$ initially and after 1000 cycle runs at a rotation rate of 1600 rpm.

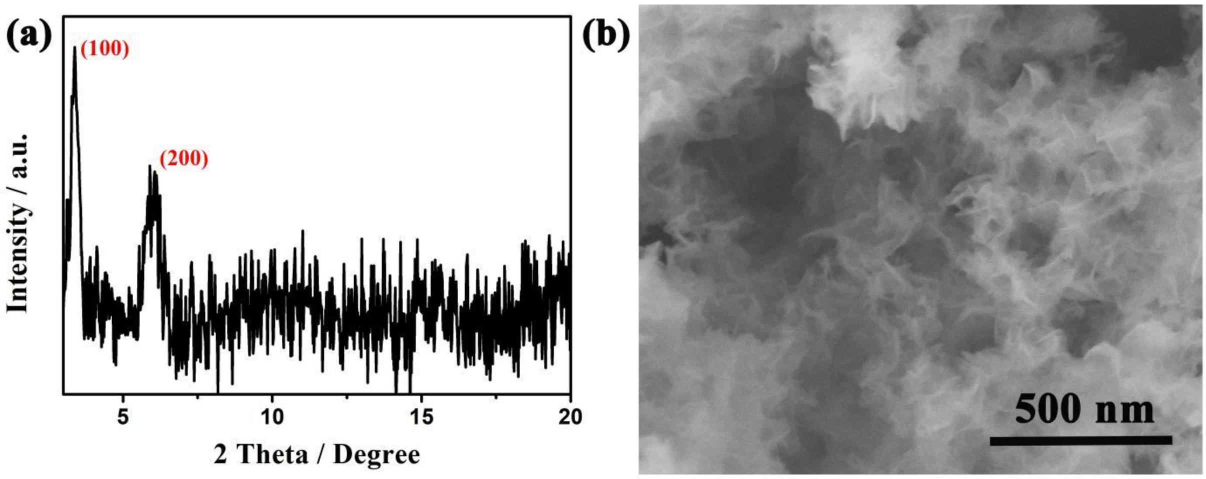

Figure S28. XRD pattern and SEM image of $Ni_3(Ni_3 \cdot HAHATN)_2$ sample after HER electrocatalytic test.
